# Supplementary material for: Multifunctional quantum dot DNA hydrogels
Source: Nat Commun. 2017 Aug 29;8:381. doi: 10.1038/s41467-017-00298-w (PMC5575008; doi:10.1038/s41467-017-00298-w)
Supplement: Supplementary file 1 — Supplementary Information [file 41467_2017_298_MOESM1_ESM.pdf]

File Name: Supplementary Information
Description: Supplementary Figures and Supplementary Tables

File Name: Peer Review File
Description:

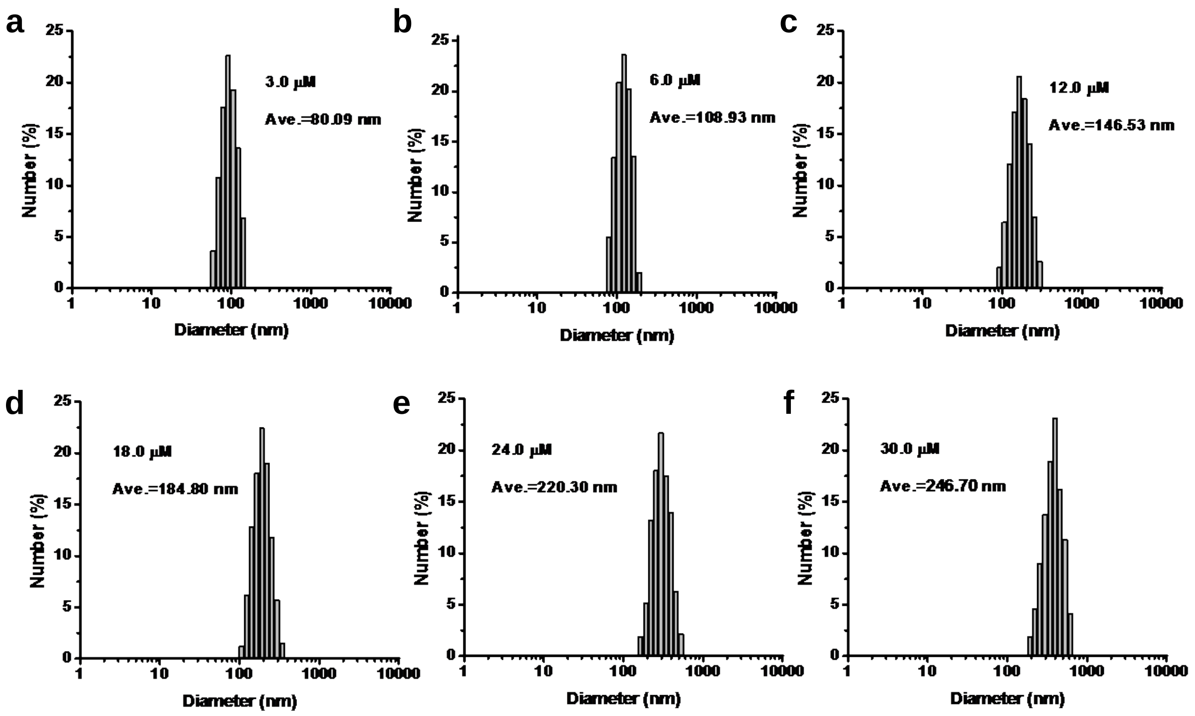

Supplementary Figure 1. Hydrodynamic size of QDHs with different initial concentration. Differential light scattering was used to estimate the size and dispersity of the QDHs.

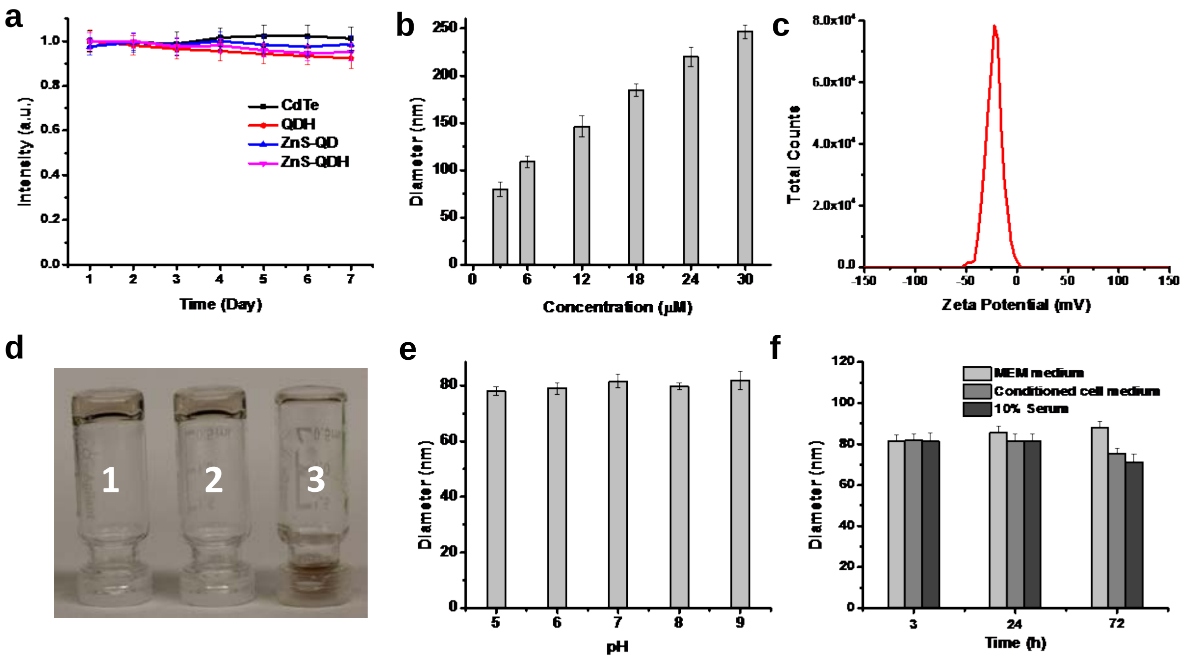

Supplementary Figure 2. QDH characterization. (**a**) The stability of the fluorescence intensity of QDs and QDH in different time. (**b**) Hydrodynamic size of QDHs with varying initial concentration. (**c**) Zeta potential of QDH analyzed by dynamic light scattering. (**d**) The temperature and enzymatic responsiveness of DNA hydrogels:(1) room temperature; (2) 37 °C for 3 h ;(3) DNase at 37 ° C for 3 h. Stabilities of QDHs monitored by luminescence and size changes at different pH after 72 h incubation (**e**) and different types of media over time (**f**). All error bars indicate s.e.m. (n=3).

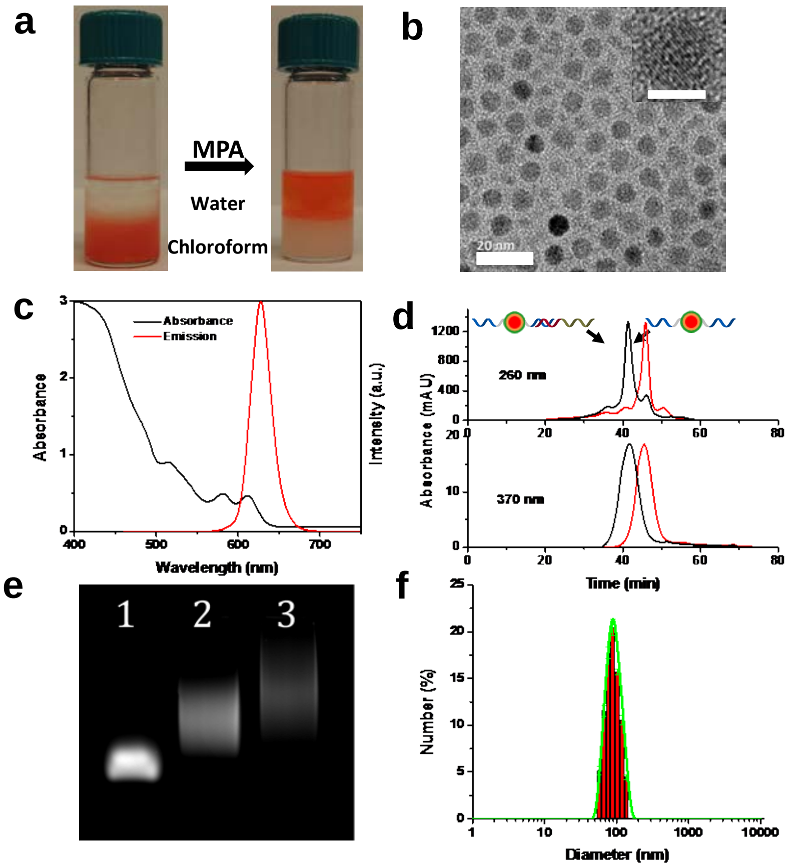

Supplementary Figure 3. Characterization of CdSe/CdS/ZnS QDs. (**a**) Red colored colloidal dispersion of CdSe/CdS/ZnS QDs undergoes the phase transfer from chloroform to water solution upon exchange of the original surface ligands with MPA. (**b**) TEM images of the CdSe/CdS/ZnS QDs (scale bars are 20 nm and 5 nm for low magnification and HRTEM images respectively). (**c**) Absorption and PL spectra of CdSe/CdS/ZnS QDs capped with MPA ligands dispersed in water solution. (**d**) High performance liquid chromatography characterization of CdSe/CdS/ZnS/DNA and the hybridization of template with complementary DNA. (**e**) Gel electrophoresis analysis of the binding of DNA to QD. Lane 1: QD; Lane 2: the mixture of QD and DNA; Lane 3, the mixture of QD and DNA with complementary DNA. (**f**) Hydrodynamic size of CdSe/CdS/ZnS/DNA hydrogels.

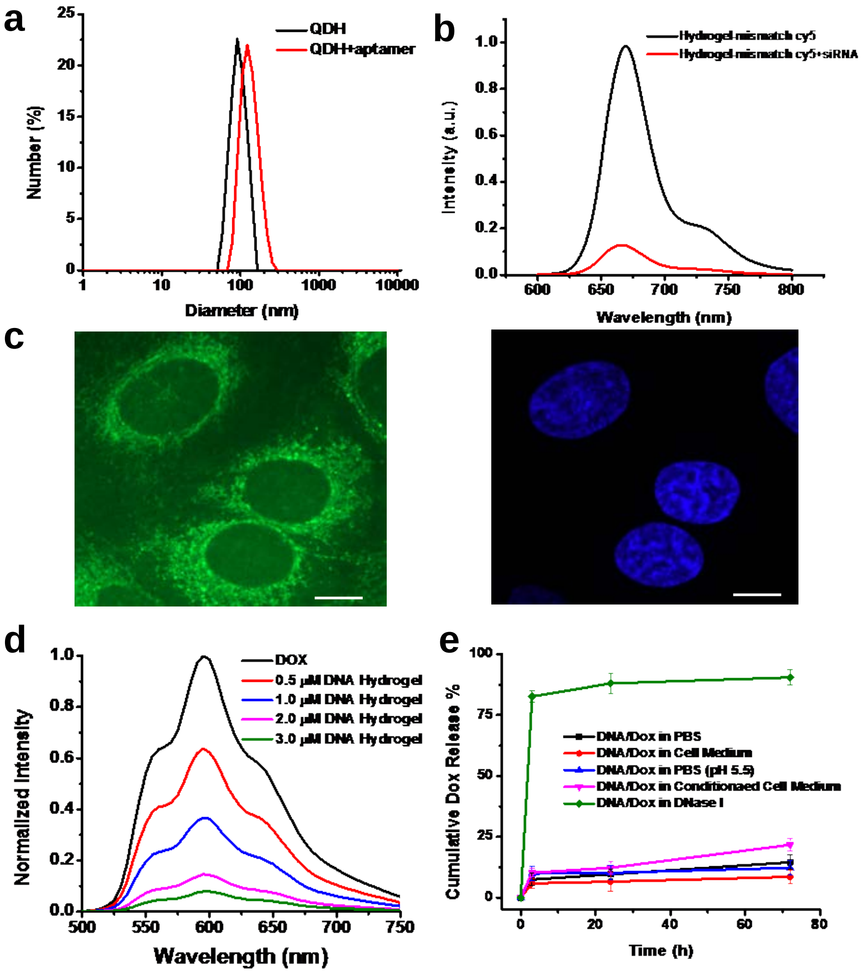

Supplementary Figure 4. Aptamer-functionalized and Dox-loaded QDHs. (**a**) Hydrodynamic size of QDHs and QDHs/aptamer. (**b**) The fluorescence intensity of before and after the competition reaction of siRNA with Cy5-labelled mismatched DNA to bind with QDHs. (**c**) Cellular accumulation of QDH-EGFR siRNA (green channel) in HeLa cells. Nuclei has been stained with Hoechst dye (blue channel). Scale bar is 5 microns. (**d**) Fluorescence spectra of Dox (50 μM) following incubation with increasing concentrations of DNA hydrogel (0-3 μM). (**e**) *In vitro* release profiles of Doxorubicin in different medium for different time at 37 °C. Error bars indicate s.e.m. (n=3).

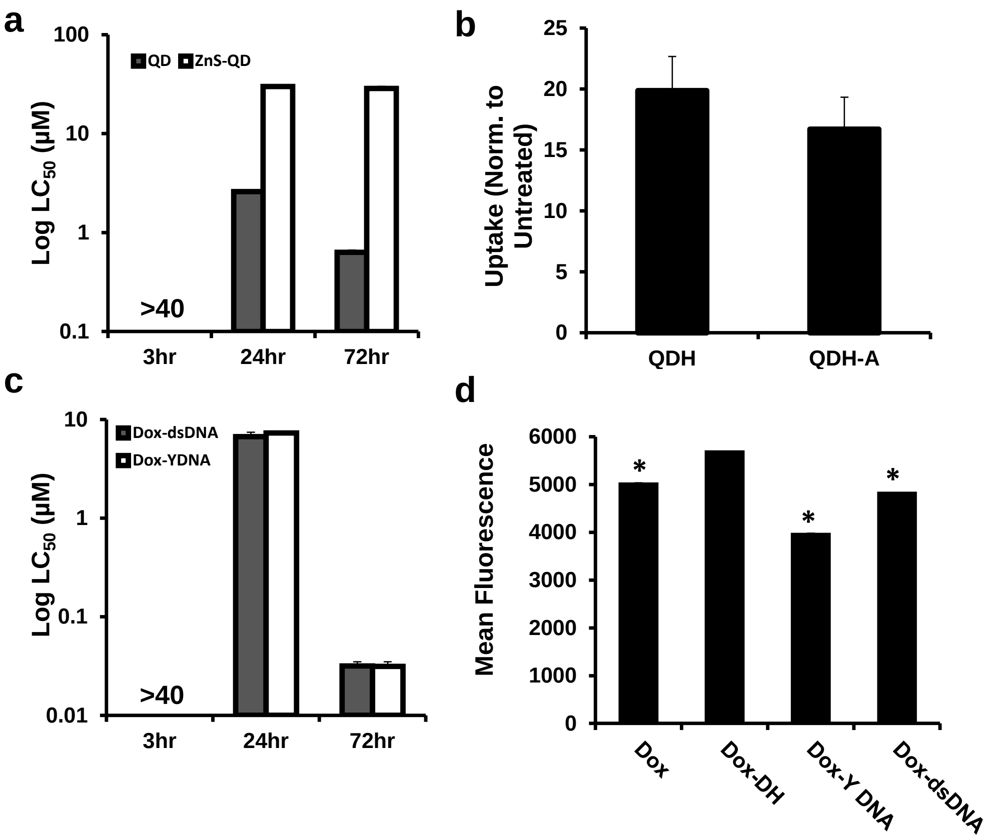

Supplementary Figure 5. Cellular toxicity of QDH with and without Dox. (**a**) Cell viability upon treatment with QD and ZnS-QD. (**b**) Measurement of cellular uptake of QDH and QDH-A in Ramos cells. (**c**) Cell viability of Dox-dsDNA and Dox-YDNA. (**d**) Comparison of potency of Dox, Dox-DH, Dox-YDNA, and Dox-dsDNA. All error bars indicate s.e.m. (n=3). One-way ANOVA vs. Dox-DH $p^* < 0.05$.

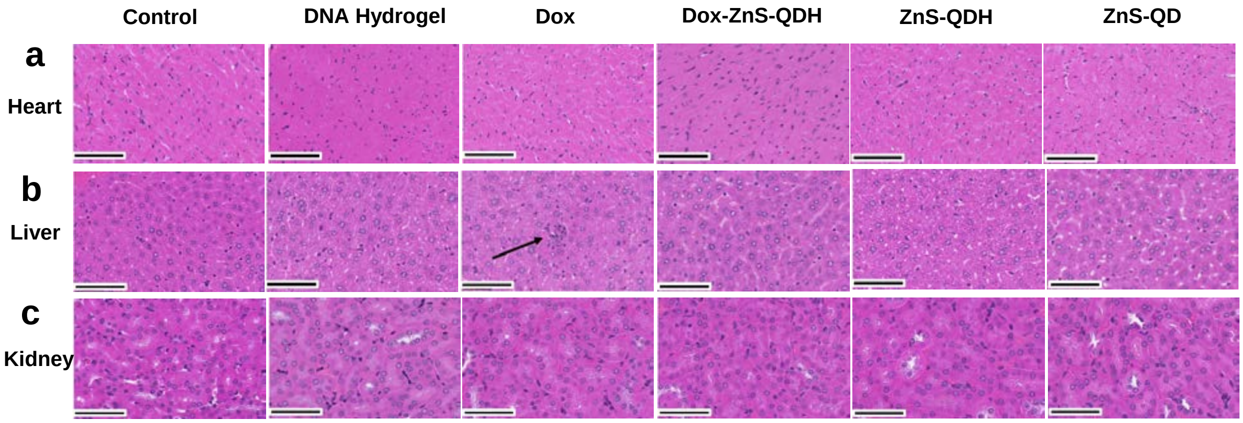

Supplementary Figure 6. H&E staining of organs collected from treatment groups of healthy mice (n=5 per group): (**a**) heart, (**b**) liver, and (**c**) kidney. Scale bar, 50 µm.

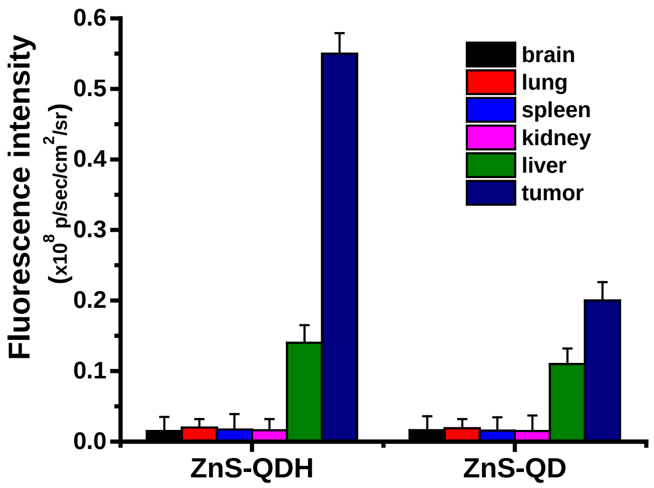

Supplementary Figure 7. Quantification of fluorescence intensity of individual organs from ZnS-QDH and ZnS-QD treated mice. All error bars indicate s.e.m. (n=5).

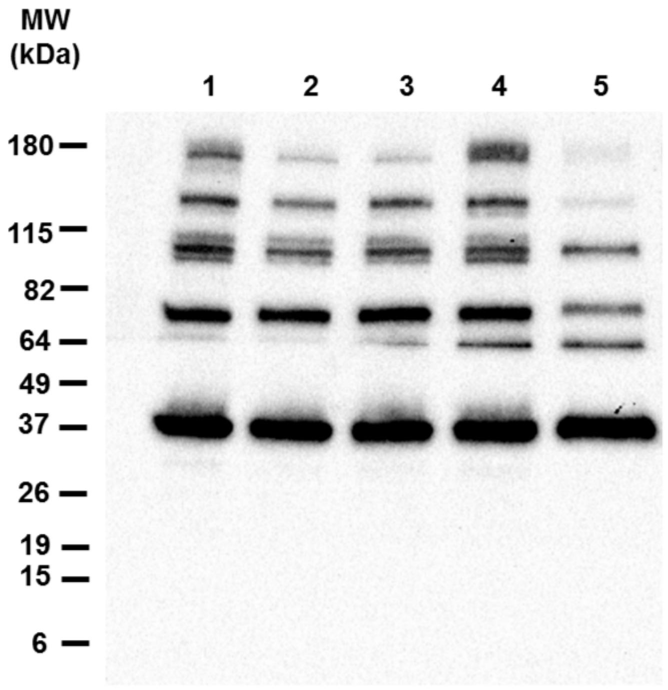

Supplementary Figure 8. Full western blot corresponding to Figure 5b. Approximate molecular weight (MW) of the protein ladder used is shown on the left side of the blot. EGFR band is depicted at ~ 175 kDa and GAPDH band at ~37 kDa. Note that the membrane (from the same gel) was cut in half to immunoblot for EGFR and GAPDH separately in their respective MW range.

Supplementary Table 1. Quantum yields for QDHs.

Quantum Dots	Quantum Yield (%)
CdTe (Green)	43.7
CdTe (Yellow)	35.6
CdTe (Red)	31.5
CdSe/CdS/ZnS	70.0

Supplementary Table 2. The swelling degree Q of the QDHs.

Hydrogels (initial concentration)	Q (%)
200 μM	538.35 ± 30.24
100 μM	341.69 ± 26.81
30 μM	186.52 ± 24.17

Supplementary Table 3. DNA and RNA sequences

Name	Sequence (5′ - 3′) (*indicate phosphorothioate linkage)
QD-DNA	GAGAGTCAATCGAAAAAAG*G*G*G*T*G*G*G*G*AAAAAAGAGAGTCAATCG
Y1	CGATTGACTCTCGCTGTCCTAACCATGACCGTCG
Y2	CGATTGACTCTCCGACGGTCATGTACTAGATCAG
Y3	CGATTGACTCTCCTGATCTAGTAGTTAGGACAGC
Y1-2	GCTGTCCTAACCATGACCGTCGCGATTGACTCTC
Y2-2	CGACGGTCATGTACTAGATCAGCGATTGACTCTC
Y3-2	CTGATCTAGTAGTTAGGACAGCCGATTGACTCTC
Aptamer	CGATTGACTCTCAAAAAATCTAACTGCTGCGCCGCCGGGAAAATACTGTACGGTTAGA
mis-cy5	Cy5-CGAGTGACTATC
siRNA sense	rGrCrArArArGrUrGrUrGrUrArArCrGrGrArArUrArGrGrUrArU
siRNA antisense	RArUrArCrCrUrArUrUrCrCrGrUrUrArCrArCrArCrArCrUrUrUrGrCAAAAATAAAAATAAAAA CGATTGACTCTC
dsDNA	CGATTGACTCTCGCTGTCCTAACCATGACCGTCGCTGATCTAGTAGCAGC
	GCTGCTACTAGATCAGCGACGGTCATGGTTAGGACAGCGAGAGTCAATCG
